# Ambulatory measurement of knee motion and physical activity: preliminary evaluation of a smart activity monitor

**DOI:** 10.1186/1743-0003-3-21

**Published:** 2006-09-13

**Authors:** James Huddleston, Amer Alaiti, Dov Goldvasser, Donna Scarborough, Andrew Freiberg, Harry Rubash, Henrik Malchau, William Harris, David Krebs

**Affiliations:** 1 Harvard Medical School Harris Orthopaedic Biomechanics and Biomaterials Laboratory Massachusetts General Hospital 55 Fruit Street, GRJ 1126 Boston, MA 02114–2696; 2 Harvard Medical School Massachusetts General Hospital Biomotion Laboratory MGH Institute of Health Professionals Charlestown Navy Yard 36 First Avenue, #223 Boston, MA 02129–4557; 3 Department of Orthopaedic Surgery Stanford University School of Medicine 300 Pasteur Drive, R-105 Stanford, CA 94305–5341

## Abstract

**Background:**

There is currently a paucity of devices available for continuous, long-term monitoring of human joint motion. Non-invasive, inexpensive devices capable of recording human activity and joint motion have many applications for medical research. Such a device could be used to quantify range of motion outside the gait laboratory. The purpose of this study was to test the accuracy of the modified Intelligent Device for Energy Expenditure and Activity (IDEEA) in measuring knee flexion angles, to detect different physical activities, and to quantify how often healthy subjects use deep knee flexion in the ambulatory setting.

**Methods:**

We compared Biomotion Laboratory (BML) "gold standard" data to simultaneous IDEEA measures of knee motion and gait, step up/down, and stair descent in 5 healthy subjects. In addition, we used a series of choreographed physical activities outside the BML to confirm the IDEEA's ability to accurately measure 7 commonly-performed physical activities. Subjects then continued data collection during ordinary activities outside the gait laboratory.

**Results:**

Pooled correlations between the BML and IDEEA knee flexion angles were .97 +/- .03 for step up/down, .98 +/- .02 for stair descent, and .98 +/- .01 for gait. In the BML protocol, the IDEEA accurately identified gait, but was less accurate in identifying step up/down and stair descent. During sampling outside the BML, the IDEEA accurately detected walking, running, stair ascent, stair descent, standing, lying, and sitting. On average, subjects flexed their knees >120° for 0.17% of their data collection periods outside the BML.

**Conclusion:**

The modified IDEEA system is a useful clinical tool for evaluating knee motion and multiple physical activities in the ambulatory setting. These five healthy subjects rarely flexed their knees >120°.

## Background

The complexity of human physical activity has made it challenging to produce a validated, accurate, and cost-effective technique to quantify activities of daily living [1-3]. The value of a sophisticated gait lab is well-established, but gait labs are expensive, require trained personnel, and may not simulate normal environments. Of the portable devices, those with accelerometers are effective in monitoring human activity when that activity is known [4-14]. These devices are attractive because they are small, non-invasive, and inexpensive. Unfortunately, many are not "smart" enough to determine the type of physical activity (e.g. stair ascent vs. walking on level ground) that the subject is performing. Pedometers are generally not sensitive to differences in stride length and are less accurate when worn by obese patients [15]. Actometers and wrist/ankle devices can provide qualitative data via "on" and "off" switches, but they are limited in their ability to record quantitative data [14,16,17]. Foot-contact monitors and electronic load transducers are problematic in their technical and practical limitations, and no reports exist in the literature regarding their accuracy in measuring human physical activity[18].

The Intelligent Device for Energy Expenditure and Activity system (IDEEA™, MiniSun, LLC), a microcomputer-based portable physical activity measurement device, allows detection of multiple gaits, limb movements, and postures (walk, run, up stairs, down stairs, stand, sit, step, jump, lie, recline, transition, etc.). It also analyzes gait, speed, distance, power, work, and energy expenditure. The IDEEA system's accuracy has been evaluated in previous investigations [18,19]. Their validation protocol required subjects to perform a series of choreographed activities. The timing of these various activities was then recorded and compared to the IDEEA. The IDEEA system was found to be accurate in measuring energy expenditure, postures and limb movements, and speed of walking and running. The original IDEEA, as described in the investigation above, was modified for our study by adding two electrogoniometers.

Various rehabilitation protocols and prosthetic knee designs assume that knee flexion as measured in laboratories reflects ambulatory knee range of motion, but this assumption, to our knowledge, has not been tested. In particular, implant manufacturers now produce "high flexion" total knee designs that may safely permit up to 150° knee flexion [20-22], but whether even healthy subjects employ these ranges of motion has not been investigated outside gait laboratories. In the present study we investigated the validity of the modified IDEEA system's ability to accurately detect physical activities and knee flexion angles compared to the Massachusetts General Hospital Biomotion Laboratory (BML). The BML permits full body analyses of kinematics and kinetics using the Selspot/TRACK data acquisition system during standing and locomotion activities, with precision and accuracy of < 1 mm position and < 1° orientation [23]. We hypothesized that 1) knee flexion angles as reported by the IDEEA would correlate with knee flexion angles as recorded by the BML and 2) the IDEEA would accurately detect activities performed during short choreographed sessions outside the BML. Validation of the modified IDEEA in healthy subjects would corroborate previous investigations, and in addition provide the error boundaries for use in determining knee flexion angles and activities of daily living in the outpatient setting. The ability to evaluate these parameters at home has numerous potential applications for patients with disorders of the musculoskeletal and neurological systems.

## Materials and methods

### Subjects

A convenience sample of 3 males and 2 females were included in this study (mean age 43.8 ± 14.5 yrs; body mass index 24.1 ± 2.9). An orthopaedic surgeon performed a detailed history and physical examination on the subjects to ensure that none of them had any orthopaedic or neurological disorders. The study group consisted of 2 orthopaedic surgeons, one real estate broker, and 2 members of the BML research team. Our institutional review board approved this study and all subjects provided written, informed consent.

### Instrumentation

#### Biomotion Lab (BML) System

**Figure 1:**
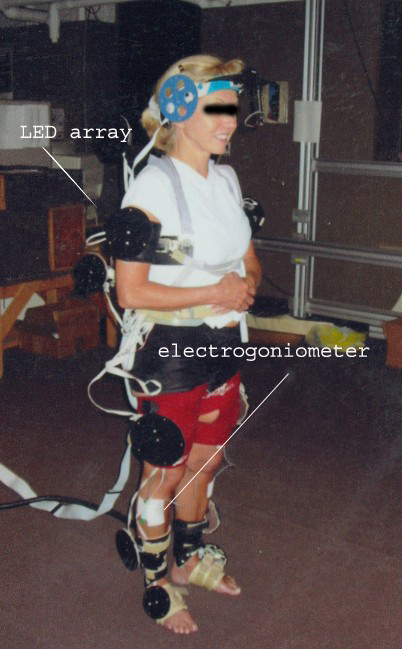
Photograph of a subject being tested in the MGH Biomotion Laboratory with both the IDEEA system and LED arrays in place.

Subjects' data were captured using a 4-camera Selspot II optoelectric light-emitting diode (LED) tracking system (Selective Electronics, Partille, Sweden) and two side-by-side Kistler piezoelectric force platforms (Kistler Instruments, Winterthur, Switzerland). LED arrays were placed on the mid-sections of 11 body segments (feet, legs, thighs, pelvis, trunk, arms and head) enabling globally referenced, 6 degrees-of-freedom (6 DOF) kinematics to be captured for each body segment (Figure 1). The LEDs were sampled at 150 Hz and filtered by using a low-pass Butterworth filter (4th order, 6-Hz cut-off, zero lag). LED array trajectories were analyzed by using SUPERTRACK^© ^software (Massachusetts General Hospital, Boston, MA) and resolved into three-dimensional (3D), 6-DOF, body segment kinematics within the 2 × 2 × 2 meter viewing volume. Subjects' anatomical data were then used to transform the global 6 DOF kinematics to 6 DOF body segment kinematics [24] Body segment mass, center of mass, and mass moment of inertia were computed from regression equations using subject-specific anatomical measurements[23] Segment angular and linear velocities and accelerations were computed by numerical differentiation of segment position data, and used with segment mass-inertial data to compute the net joint torques based on the Newtonian inverse dynamic approach.

### IDEEA System

**Figure 2:**
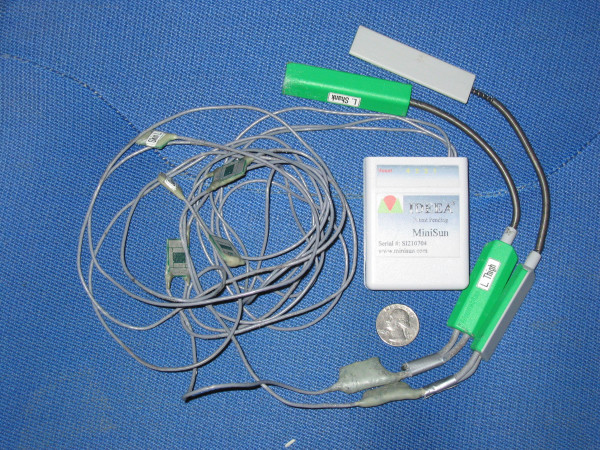
Photograph of the modified IDEEA system. The two electrogoniometers (green) were added to the system by MiniSun, LLC for our study.

**Figure 3:**
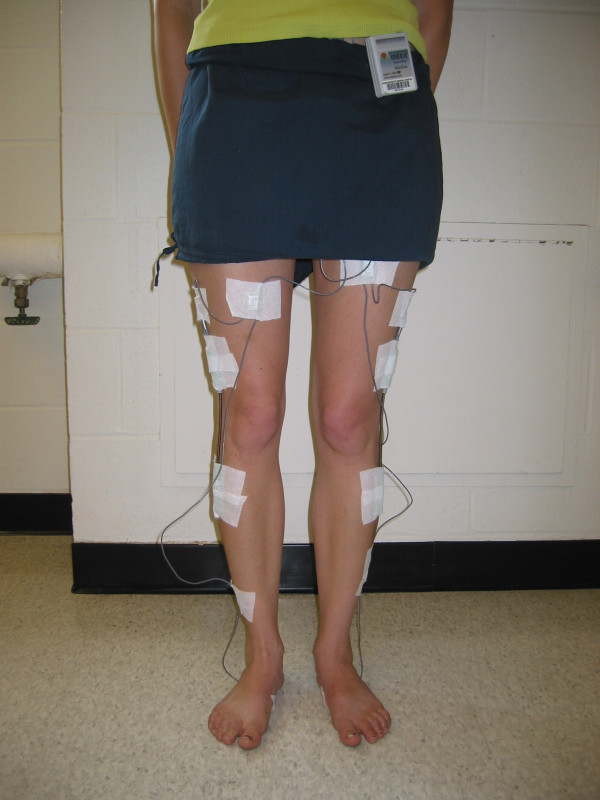
Photograph (front view) of the modified IDEEA system being worn by a patient.

**Figure 4:**
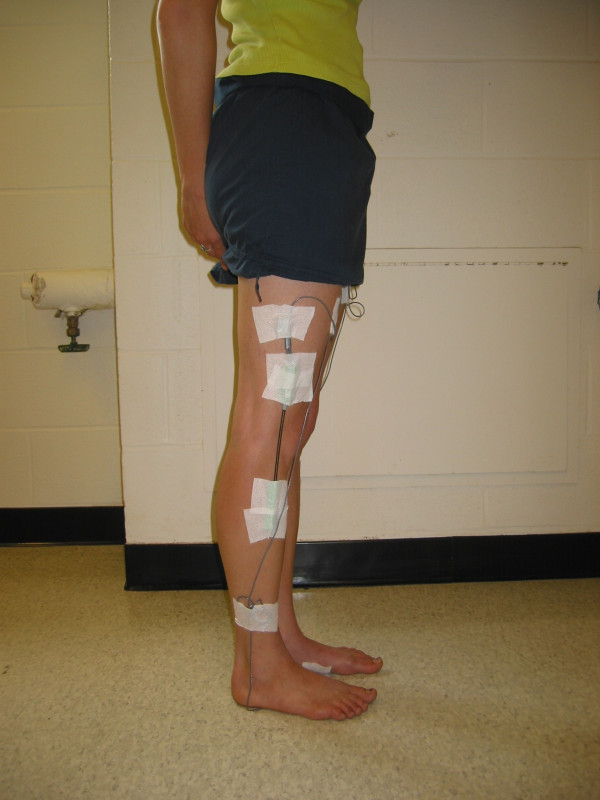
Photograph (side view) of the modified IDEEA system being worn by a patient.

The Intelligent Device for Energy Expenditure and Activity identifies multiple human physical activities based on limb movements, postures, transitions, and gaits; it quantifies these physical activities by type, duration, intensity, and expended energy. The mean and standard deviation for 18 measured parameters are calculated for right foot, left foot, and both feet. The device includes 5 sensors (each 16 × 14 × 4 mm) (Figure 2). The sensors measure angles and acceleration of body segments in 2 orthogonal directions. One sensor was placed in the midline approximately 2 cm distal to the sternal notch. One sensor was placed on the plantar aspect of each foot, proximal to the metatarsal heads. One sensor was placed on the anterior surface of each thigh at the mid-femur level (Figures 3 and 4). Hypoallergenic adhesive tape was used to secure the sensors to the skin. Each sensor was placed with the proper side against the skin and in line with the longitudinal axis of the body segment. Output signals travel via 2 mm cables to a 33 MHz, 32-bit ARM microprocessor (ARM, Cambridge, UK) housed in a 7 × 5.4 × 1.7 cm plastic box. The box weighs 59 grams and is worn on one's belt. Flash memory allows recording of the data during activities of daily living without loss of data even if unexpected power failure occurs. The device operates using a single AA alkaline battery and consumes approximately 0.045 watts during operation.

At our request, knee electrogoniometers (Penny+Giles™, Biometrics Ltd., United Kingdom) were added to the system by Minisun, LLC. The electrogoniometers measure knee flexion angles and were calibrated by MiniSun, LLC. One electrogoniometer was placed on the lateral surface of each knee, in line with the anatomic axes of the femur and tibia, and fixed to the skin with hypoallergenic tape with the knee in 0° extension (as measured by a conventional goniometer). Data collected is downloaded to a personal computer via a USB connection and a software interface (provided by Minisun, LLC, the software also interprets the sensor output and determines activity type and other variables). With the addition of the electrogoniometers, the IDEEA can operate continuously for up to 48 hours and can store greater than 60 million data points at 32 Hz. The output of the modified IDEEA software gives the knee flexion angles and, simultaneously, identifies activities (walking, sitting, standing, running, stairs, recline, transition, etc.) in charts, tables, and "movie-like" animation. For a specific time interval, as short as 1/32 second, it calculates the time that each different physical activity was performed.

### Protocol

An experienced member of the research team applied the modified IDEEA to the subject; another team member confirmed proper placement. Prior to collecting data, the device was calibrated with the subject sitting in a chair with the hips and knees in 90° of flexion and the ankles in neutral dorsiflexion. The proper electrogoniometer position was again confirmed by a conventional goniometer. Small differences in time between the clocks and knee angles in the IDEEA system and in the BML were noted at the time of calibration and corrected during data analysis.

Five subjects' data were collected using the BML and the IDEEA simultaneously. Subjects performed each task at least twice with at least one practice trial prior to data collection. Gait trials consisted of the subjects walking at their preferred pace along a 10-meter walkway. Stair descent included descending a four-step modular staircase of outdoor height (18 × 28 cm) without railings. Stepping up and down a 7.5 cm height stair was performed at a metronome cadence of 100 beats per minute for 30 sec [25].

To simulate a more realistic clinical scenario, we implemented a second protocol requiring subjects to perform a series of common activities for 30-second intervals outside the testing conditions in the BML (no LED arrays). These activities included: running, walking, sitting, ascending stairs, descending stairs, standing, and lying. The exact time that these activities occurred was recorded by a member of the research team and then compared to the data generated by the IDEEA system.

After testing in the BML, the LED arrays were removed and subjects changed back into their street clothes. They then departed with the IDEEA in place and with instructions to perform their daily activities as usual. We requested that each subject wear the device at least 7 hours and for up to 24 hours. Subjects were permitted to exercise but they were not allowed to shower or bathe with the device in place. The majority of data collection outside the BML occurred while the subjects were at work. One orthopaedic surgeon wore the IDEEA during a day in the operating theatre. The other orthopaedic surgeon wore the device while seeing patients in clinic. The BML researchers spent the majority of their data collection period working on a computer at a desk. The real estate broker transported clients by car to view residential property.

### Data Analysis

Data were processed and analyzed after testing each subject. The same time interval of BML and IDEEA data was analyzed for the three specific physical activities (gait, stepping, and stair descent). Pearson correlation was used to compare the knee flexion angles recorded by the BML and the IDEEA. Choreographed activities outside the BML were checked against the reporting of the IDEEA for the known time intervals.

## Results

**Figure 5:**
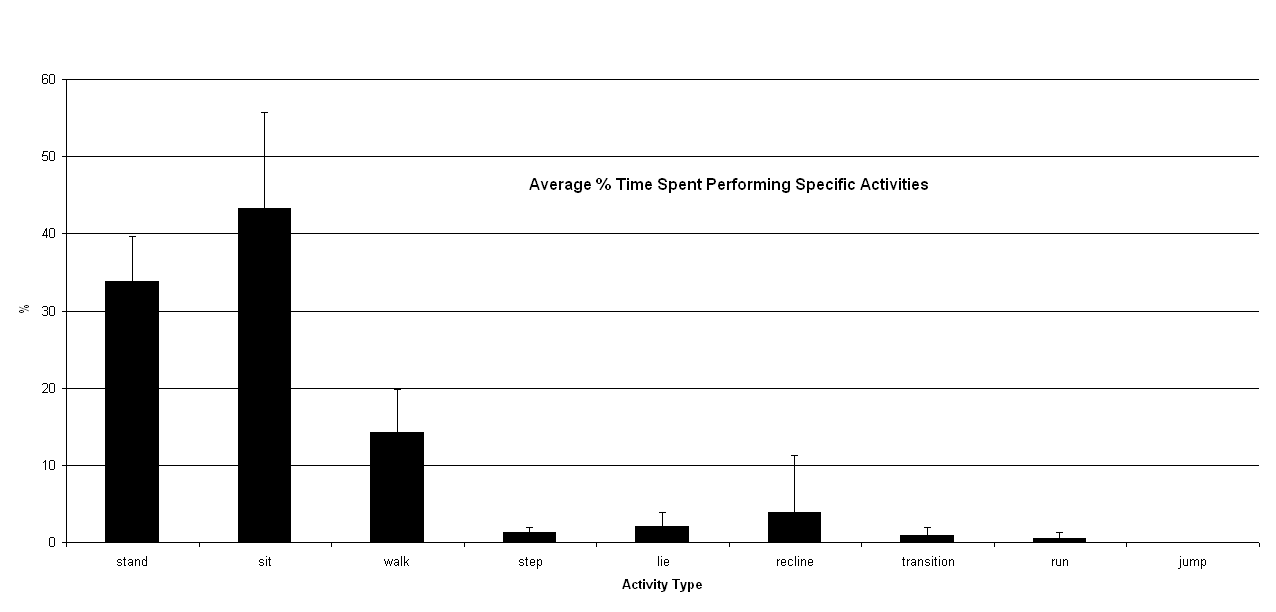
This histogram shows the average time (%) that the 5 subjects spent performing various activities during their data collection periods outside the BML.

The subjects used the IDEEA (including time during testing in the BML) for an average of 17.4 +/- 9.7 hours (range, 7.5–33 hours). Two subjects wore the device overnight while sleeping. Figure 5 summarizes the various activities performed by all subjects while awake. Subjects took an average of 8,441 +/- 4,785 steps (range, 4,369–14,715 steps) per session. Table 1 quantifies the various activity parameters recorded by the IDEEA system during each subject's entire data collection period.

**Table 1:** General Activity Parameters Measured by IDEEA

**Subject**	**Session Duration (hours)**	**Steps (#)**	**Distance (km)**	**Speed (m/min)**	**Energy Expenditure (kcal/minute)**
1	11.5	5670	4.4	9.9	2.7
2	7.4	4997	3.8	16.0	2.8
3	18.7	4369	4.0	5.6	2.4
4	16.1	14,715	11.0	16.3	2.2
5	33	12,452	9.3	10.5	1.8
mean	17.4 +/- 9.7	8441 +/- 4785	6.5 +/- 3.4	11.7 +/- 4.5	2.4 +/- 0.4

**Figure 6:**
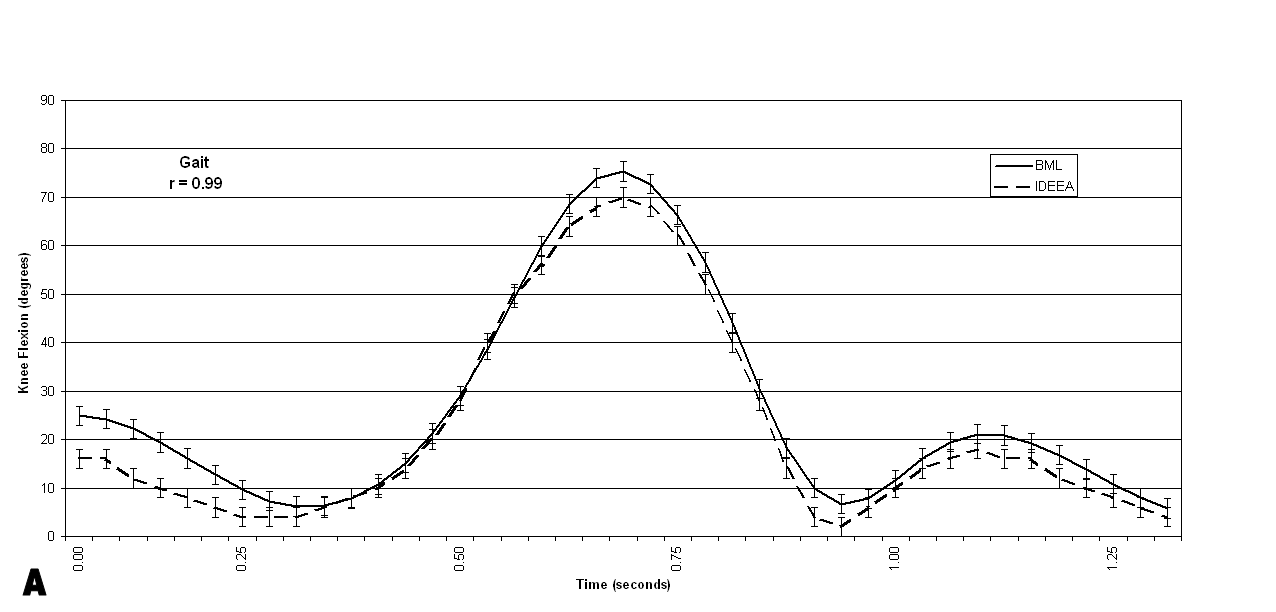
This graph shows the knee flexion angles, for one subject, recorded simultaneously by the IDEEA and the BML during 3 trials of gait.

**Figure 7:**
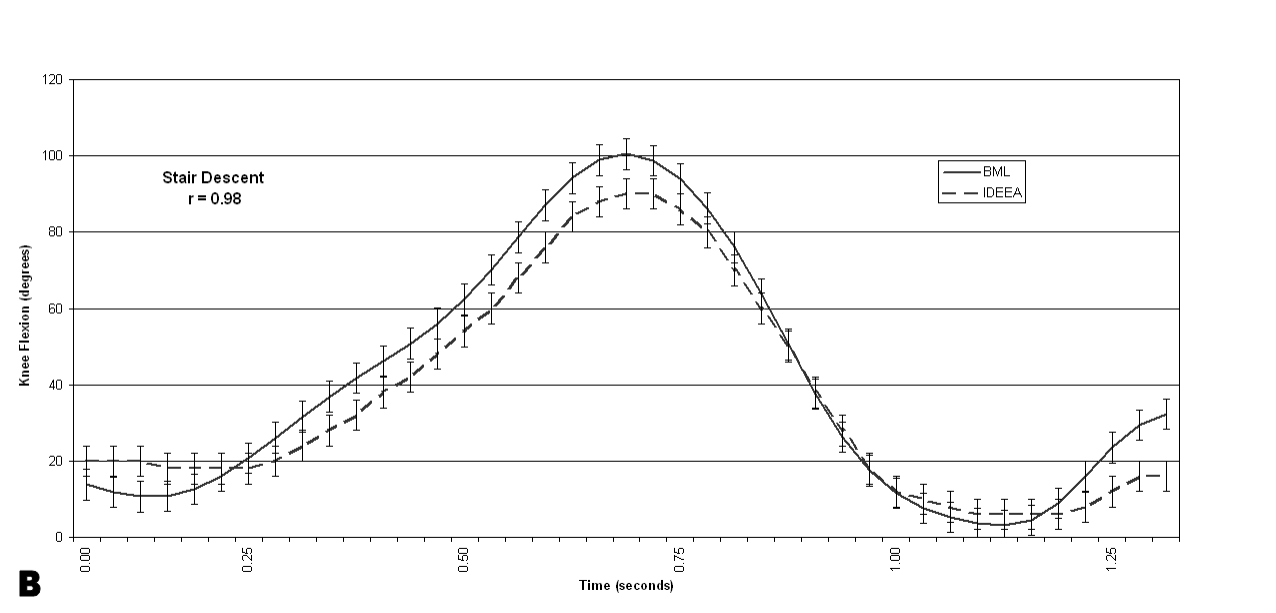
This graph shows the knee flexion angles, for one subject, recorded simultaneously by the IDEEA and the BML during 3 trials of stair descent.

**Figure 8:**
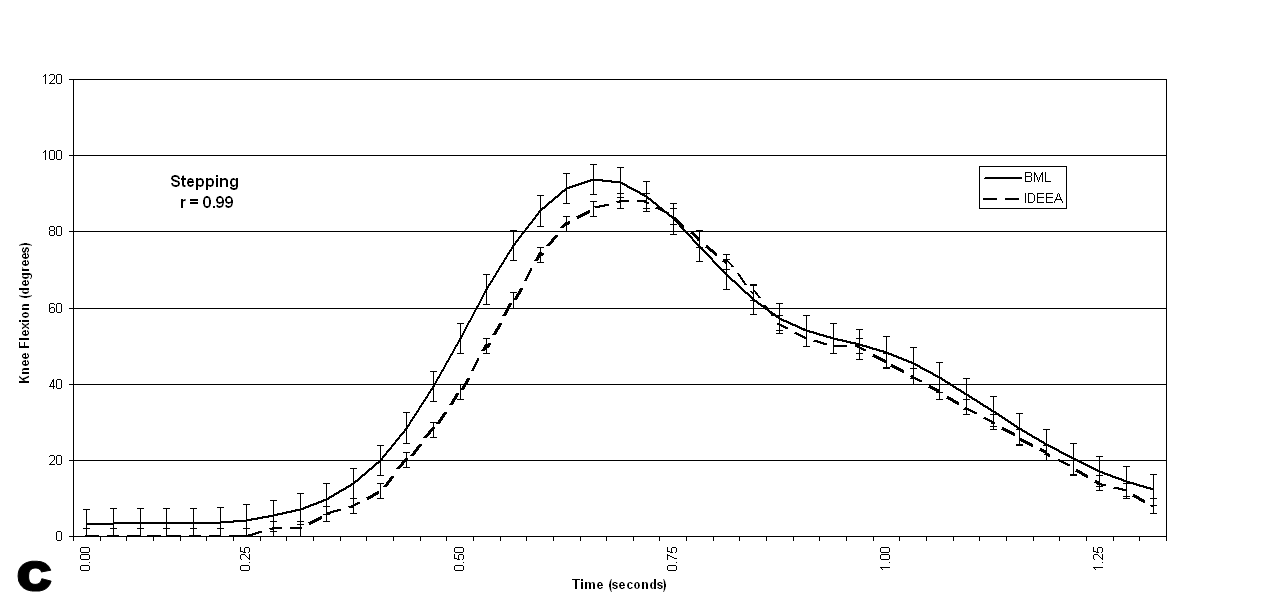
This graph shows the knee flexion angles, for one subject, recorded simultaneously by the IDEEA and the BML during 3 trials of stepping up and down on a single step.

**Figure 9:**
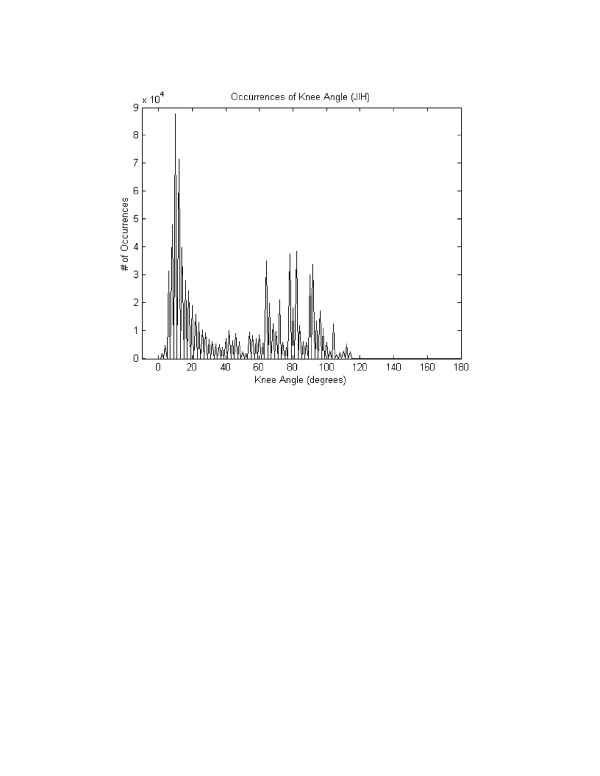
This graph shows the number of times that one subject (JH) reached various knee flexion angles during his data collection period outside the BML.

The pooled correlations between the BML and the IDEEA system knee flexion angles were .98 +/- .01 for gait, .98 +/- .02 for stair descent, and .97 +/- .03 for step up/down (Figures 6, 7, 8). Four of 5 subjects flexed their knees >120° at any time during their data collection periods. Two subjects recorded knee flexion >160°, both during sitting with their foot underneath their contralateral buttock. Time spent at >120° of knee flexion averaged 58 +/- 39 seconds (range, 0–267 seconds). This time spent at knee flexion >120° represented, on average, 0.17 % of each subject's testing session. Figure 9 shows the number of occurrences of various knee flexion angles, for one subject, during the data collection period outside the BML.

In testing during the choreographed sessions outside the gait laboratory, the IDEEA accurately reported activity for all 5 subjects in all trials (Table 2). While testing in the BML protocol, the IDEEA system accurately identified the gait trial in all five subjects. It correctly identified stair descent in three of the five subjects. For the stair descent trials of the other two subjects, the IDEEA incorrectly reported their activity as "walking". The IDEEA identified stepping up/down correctly in only two of the five subjects. It incorrectly reported "walking" during the step up/down trials for the other three subjects (Table 3).

**Table 2:** Accuracy of Activity Identification Outside BML

**Known Activity**	**Activities by Subject as Reported by IDEEA**				
	
	1	2	3	4	5
walk	walk	walk	walk	walk	walk
run	run	run	run	run	run
stair ascent	step up	step up	step up	step up	step up
stair descent	step down	step down	step down	Step down	step down
lie	lie	lie	lie	lie	lie
stand	stand	stand	stand	stand	stand
sit	sit	sit	sit	sit	sit

**Table 3:** Activity as Reported by IDEEA during BML Protocol

**Subject ID**	**Gender**	**Step Trial**	**Gait Trial**	**Stair Descent Trial**
1	M	walk	walk	step down
2	M	walk	walk	step down
3	F	step up	walk	walk
4	M	step up	walk	walk
5	F	walk	walk	step down

## Discussion

The frequency that healthy subjects use deep knee flexion outside the laboratory setting is currently unknown. We sought to validate the modified IDEEA system by using the Selspot/TRACK data acquisition system in a laboratory and in subjects' natural environments. In the laboratory, we limited our examination to knee motion and 3 activities in 5 healthy subjects, as it is these data that hold the most relevance to prosthetic device research. Our results indicate that the IDEEA system, when compared to the gold-standard gait laboratory, is able to accurately report knee flexion angles. During BML testing it was able to accurately report gait, but it was less accurate in recording stair descent and step up/down. However, during testing outside the BML, the IDEEA accurately identified walking, running, standing, sitting, stair ascent, stair descent, and lying in the five subjects.

The inability of the IDEEA system to accurately detect stair descent and step up/down during laboratory testing may be due to the limitations inherent in our protocol. The size of the data collection area restricts both the activity type and duration of activity that can be evaluated. This size constraint limits our stair model to 4 steps. MiniSun states that 4 steps are too few to allow the IDEEA to confirm this activity. We know that we diminished the estimate of accuracy by limiting the maximum time available for activity detection. However, this was done in an effort to perform a highly standardized data analysis, as has previously been the basis for prosthetic knee design. Moreover, during the stepping protocol, subjects stepped forwards and backwards, a task IDEEA is not currently designed to detect.

Our results of testing for activity identification outside the BML protocol corroborate previous investigations [18,19]. In their validation of the IDEEA system, the authors used a timed protocol of specific activities to measure postures, limb movements, and jumping. They evaluated stair ascent and descent by timing subjects on the stairs at two different speeds. In combining the IDEEA with the flexible electrogoniometers, we have created a tool capable of determining, among others, the amount of knee flexion needed for activities that are commonly-performed outside the gait laboratory.

The pooled correlations between IDEEA and BML knee angles during step up/down, gait, and descending stairs ranged from .93 to .99. These data suggest that the IDEEA accurately measures knee motion during these three activities. The electrogoniometers proved to be durable, as none of the devices failed to collect data after the subjects exited the gait laboratory.

The data recorded by the modified IDEEA system confirm that some patients flex their knee >120° and that the system is capable of recording deep knee flexion angles up to 160°. Four of 5 subjects flexed their knee > 120° during routine activities. Two subjects flexed their knees >160° while sitting on a chair with their foot curled under the contralateral buttock. On average, these five subjects spent 0.17 % of their testing session with their knees in >120° of knee flexion. These data must be interpreted cautiously, as we only tested 5 subjects who performed jobs that do not regularly require deep knee flexion. The subjects may have used knee flexion >120° more often if they had been evaluated on a weekend or holiday.

## Conclusion

We found that the modified IDEEA system, compared to the Selspot/TRACK data acquisition system in the MGH BML, accurately reported healthy subjects' knee range of motion. The IDEEA system was also able to accurately detect walking, running, standing, sitting, stair climbing, stair descent, and lying during choreographed activities outside the BML. The results of the present study, in conjunction with previous reports, support the use of the modified IDEEA system in the outpatient setting. In the future, we plan to use the IDEEA system to evaluate knee motion and frequency and duration of activities of daily living in patients who have had total knee arthroplasty. This approach may eventually allow for the assessment of surgical outcomes for different prosthetic designs.

## Abbreviations

IDEEA Intelligent Device for Energy Expenditure and Activity

BML Biomotion Laboratory

## Competing interests

The author(s) declare that they have no competing interests.

## Authors' contributions

JH developed the ideas discussed in this paper, recruited subjects, performed the experiments, analyzed the data, and drafted the manuscript under the guidance of AF, HR, HM, WH, and DK. DG and DS performed the experiments, analyzed the data, and assisted in revising the manuscript. AF, HM, and HR assisted in revising the manuscript. WH conceived the study and assisted in revising the manuscript. DK conceived the study, recruited subjects, performed the experiments, supervised the BML experiments, and assisted in analyzing the data and revising the manuscript. All authors read and approved the final manuscript.
